# Metal-free aerobic oxidations mediated by *N*-hydroxyphthalimide. A concise review

**DOI:** 10.3762/bjoc.9.146

**Published:** 2013-07-02

**Authors:** Lucio Melone, Carlo Punta

**Affiliations:** 1 Department of Chemistry, Materials, and Chemical Engineering “Giulio Natta”, Politecnico di Milano, Piazza L. Da Vinci 32, Milano 20131, Italy; 2 INSTM, National Consortium of Materials Science and Technology, Local Unit Politecnico di Milano, Italy

**Keywords:** autoxidation, free-radicals, metal-free, molecular oxygen, *N*-hydroxyphthalimide

## Abstract

Since the beginning of the century, *N*-hydroxyphthalimide and related compounds have been revealed to be efficient organocatalysts for free-radical processes and have found ample application in promoting the aerobic oxidation of a wide range of organic substrates. When combined with different co-catalysts, they are activated to the corresponding *N*-oxyl radical species and become able to promote radical chains, involving molecular oxygen, directly or indirectly. Most of the examples reported in the literature describe the use of these *N*-hydroxy derivatives in the presence of transition-metal complexes. However, eco-friendly standards, including the demand for highly selective transformations, impose the development of metal-free processes, especially for large-scale productions, as in the case of the oxygenation of hydrocarbons. For this reason, many efforts have been devoted in the past decade to the design of new protocols for the activation of *N*-hydroxy imides in the presence of nonmetal initiators. Herein we provide a concise overview of the most significant and successful examples in this field, with the final aim to furnish a useful instrument for all scientists actively involved in the O_2_-mediated selective oxidation of organic compounds and looking for environmentally safe alternatives to metal catalysis.

## Introduction

The development of efficient and cheap catalytic systems for the selective oxidation of organic substrates under mild and environmentally benign conditions represents one of the major challenges in organic synthesis [1]. In this context, the replacement of traditional oxidants, often used in stoichiometric amounts, with molecular oxygen is mandatory in order to improve the beneficial impact of selective oxidation on industrial chemistry [2–5]. Nevertheless, classical autoxidation is usually very slow at low temperatures, and catalysis is required to activate O_2_. Transition-metal salts are particularly effective for this scope [6], but their use is often detrimental for the selectivity of the process and they would not meet the standards of “green chemistry”. An alternative catalytic route is based on the use of *N*-hydroxy imides (NHIs), and in particular *N*-hydroxyphthalimide (NHPI), which have found ample application as ideal catalysts for the aerobic oxidation of organic substrates [7–11].

**Scheme 1:**
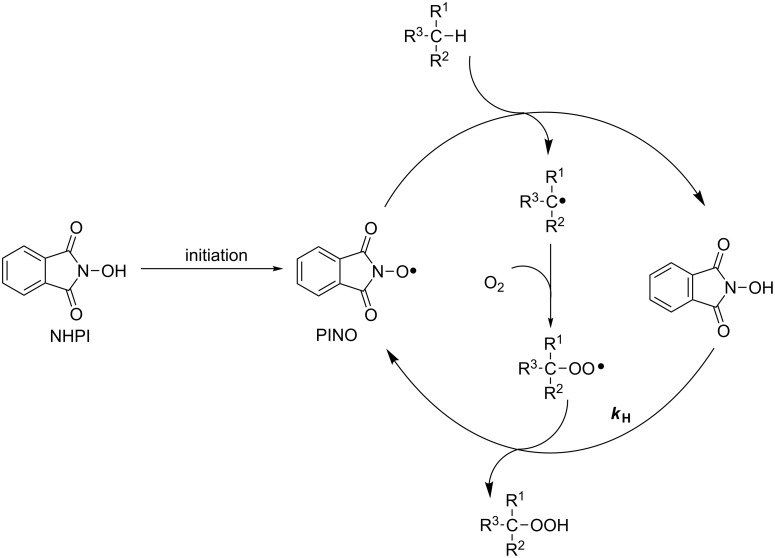
Catalytic role of NHPI in the selective oxidation of organic substrates.

NHPI acts as a precursor of the phthalimide *N*-oxyl (PINO) radical, which is the effective catalyst promoting hydrogen-abstraction processes (Scheme 1).

The reactivity of NHPI and PINO is related to the bond dissociation energy (BDE) of the O–H group, which was estimated at 88.1 kcal/mol [12]. This value is similar to the BDE of O–H in hydroperoxides, suggesting that the faster reactivity of PINO compared to peroxyl radicals should be attributed to an enhanced polar effect involved in the hydrogen abstraction by this nitroxyl radical [13]. Furthermore, NHPI also behaves as a relatively good hydrogen donor even at low temperatures (*k*_H_ = 7.2 × 10^3^ M^−1^s^−1^) [12], trapping peroxyl radicals before they undergo termination.

PINO generation represents the key step of the overall process. Many transition metal salts and complexes have been successfully used as co-catalysts for NHPI activation. However, once again their use should be avoided in order to improve the sustainability of the process. For this reason, in the past decade several efforts have been devoted to the development of catalytic systems for the metal-free activation of NHIs under mild conditions. With an overview on the results reported in the literature in the past decade, we aim to describe herein the most significant examples related to the selective oxidation of organic molecules with molecular oxygen, catalyzed by NHIs in the presence of nonmetal cocatalysts. After briefly describing the role of classical radical initiators obtained by thermal decomposition, we will focus on some intriguing redox systems, including nitric oxides, laccase, quinones and aldehydes, which allow operation under very mild conditions, offering efficient alternative solutions to the classical autoxidation processes, especially in the field of the selective oxygenation of hydrocarbons.

## Review

### Radical initiation by thermal decomposition

Thermal decomposition of peroxides and azo-compounds is a well-known technique generally used to generate radicals in solution. Ishii and co-workers widely investigated the key role of these initiators in promoting the formation of PINO, performing several selective transformations under aerobic or anaerobic conditions.

**Scheme 2:**
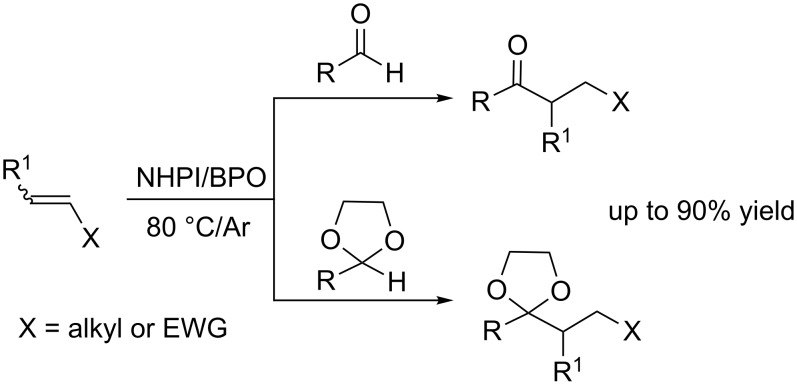
Radical addition of aldehydes and analogues to alkenes.

The combination of NHPI with tiny amounts of dibenzoyl peroxide (BPO) under an atmosphere of argon led to the hydroacylation of simple alkenes by addition of acyl radicals derived from aldehydes and masked aldehydes, such as 1,3-dioxolanes (Scheme 2) [14–15].

The mediating role of NHPI allowed this approach to be extended to electron-deficient olefins [15], preventing the polymerization that easily occurs with these substrates in the presence of radical initiators.

**Scheme 3:**
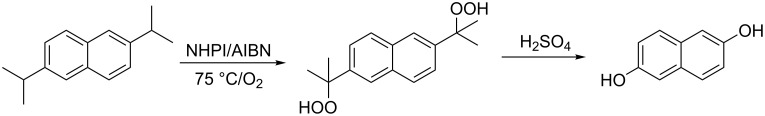
NHPI/AIBN-promoted aerobic oxidation of 2,6-diisopropylnaphthalene.

Azo-initiators were mainly employed for the synthesis of phenol derivatives by aerobic oxidation of isopropyl aromatics. For example, the oxidation of 2,6-diisopropylnaphthalene with air in the presence of α,α’-azobisisobutyronitrile (AIBN) and NHPI, followed by decomposition with sulfuric acid of the dihydroperoxide oxidation product, gave the corresponding 2,6-naphthalenediol in 92% yield (Scheme 3) [16].

As expected, the replacement of AIBN with a transition-metal complex (Co(OAc)_2_) resulted in a collapse of selectivity due to the redox decomposition of the hydroperoxides. This catalytic system was also applied to the oxygenation of 1,3,5-triisopropylbenzene [17]. However, in this case the conversion of all isopropyl groups was far from being reached, with mono- and di-phenols being the major products, while the yield in benzene-1,3,5-triol was close to 1%.

**Scheme 4:**
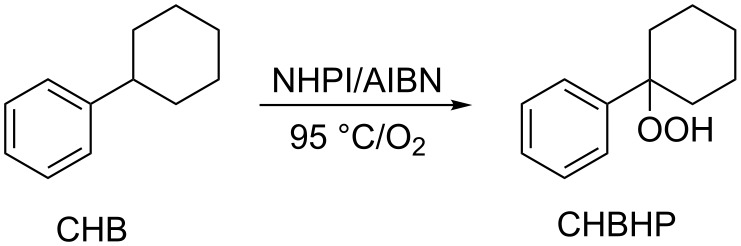
NHPI/AIBN-promoted aerobic oxidation of CHB.

Sheldon and co-workers tested several commercially available azo-compounds (including AIBN and 2,2’-azobis(2,4-dimethylvaleronitrile) (AMVN)) as radical initiators at different temperatures for the NHPI-catalyzed oxidation of cyclohexylbenzene (CHB) to the corresponding hydroperoxides, finding a high selectivity in the oxygenation at the 1-position (CHBHP, Scheme 4) [18]. The selectivity decreased rapidly with increasing conversion and temperature. The use of CHBHP itself as radical initiator in place of azo-compounds required higher temperatures (100–120 °C), leading to conversions up to 20%, but to the detriment of the selectivity (72%).

**Scheme 5:**
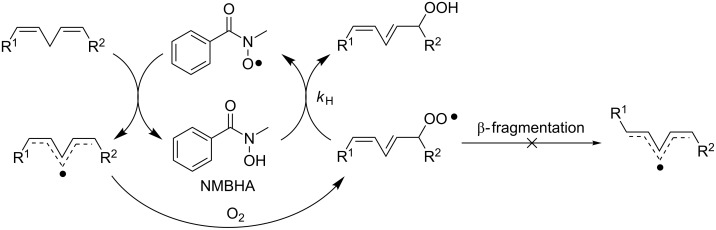
NMBHA/MeOAMVN promoted aerobic oxidation of PUFA.

An analogous catalytic system was employed by Punta et al. [19] for the selective peroxidation of polyunsaturated fatty acids (PUFA), a transformation of high biological interest [20]. The combination of *N*-methylbenzohydroxamic acid (NMBHA) with 2,2’-azobis(4-methoxy-2,4-dimethylvaleronitrile) (MeO-AMVN), an azo initiator that decomposes at physiological temperatures 15 times faster than the corresponding AMVN, allowed the *trans,cis* conjugated hydroperoxides to be obtained exclusively and in good yields by direct oxygenation of the corresponding lipids. In fact, due to its ideal O–H BDE value (79.2 kcal/mol [12]), NMBHA turned out to be a better candidate than NHPI to act both as autoxidation catalyst, promoting the hydrogen abstraction from the bisallylic C–H position of the fatty esters by means of the corresponding amidoxyl radical, and as a good hydrogen donor (*k*_H_ = 1.2 × 10^5^ M^−1^s^−1^ [19]) trapping *trans,cis* peroxyl radicals before they underwent β-fragmentation (Scheme 5).

**Scheme 6:**
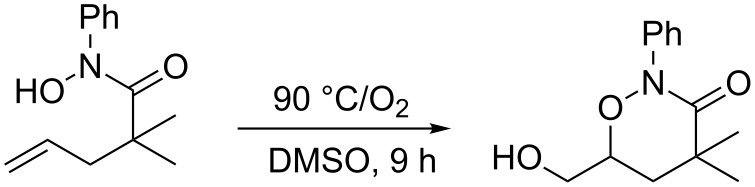
Alkene dioxygenation by means of *N*-aryl hydroxamic acid and O_2_.

In accordance with this free-radical mechanism, Schmidt and Alexanian more recently reported the aerobic dioxygenation of alkenyl *N*-aryl hydroxamic acids (Scheme 6) [21]. In this case dioxygenation proceeded under 1 atmosphere of O_2_ alone, without any additional initiator being required.

### Radical initiation by redox processes

#### Nitrogen oxides and nitric acid

**Scheme 7:**
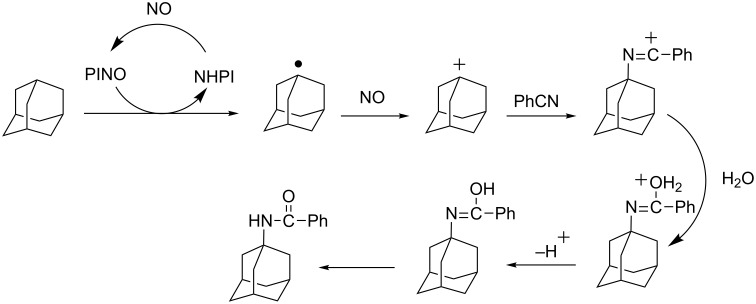
NHPI-catalyzed reaction of adamantane under NO atmosphere.

The PINO activation by means of nitric oxide was first reported by Ishii et al. in 1997 [22]. By reacting adamantane in a mixed solvent of benzonitrile and acetic acid under an atmosphere of NO and in the presence of catalytic amounts of NHPI he observed the formation of 1-*N*-adamantylbenzamide as a principal product (Scheme 7), while when operating under the same conditions but in the presence of molecular oxygen, 1-nitroadamantane was achieved in good yields.

Moreover, by simply moving into acetonitrile, they observed the selective oxygenation of phthalane to the corresponding phthalaldehyde in 80% yield [23]. In both cases the reaction proceeds via the formation of a carbocation intermediate (Scheme 7).

**Scheme 8:**
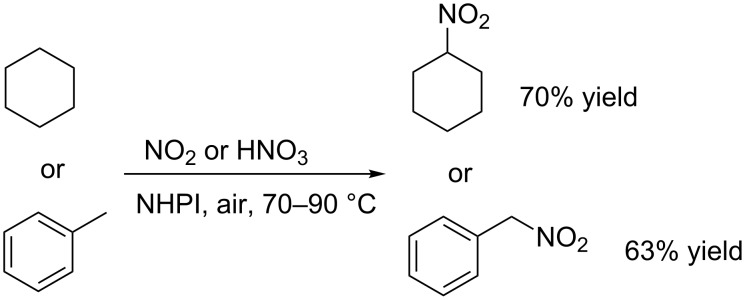
Nitration of alkanes and alkyl side-chains of aromatics.

The same research group also reported the efficient air-assisted nitration of alkanes [24] and alkyl side-chain aromatic compounds [25] by nitrogen dioxide and nitric acid, under NHPI catalysis (Scheme 8).

**Scheme 9:**
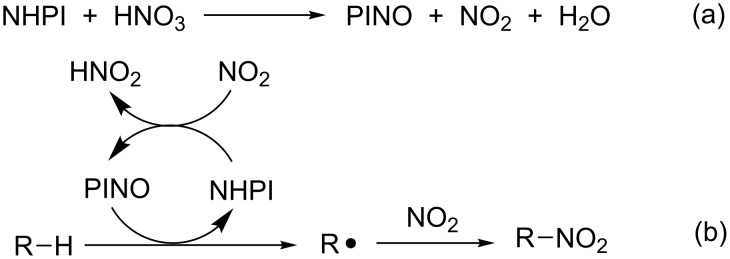
Radical mechanism for the nitration of alkanes catalyzed by NHPI.

Both HNO_3_ and NO_2_ are able to promote the formation of PINO according to path (a) and (b) reported in Scheme 9. These initiation steps lead to the formation of HNO_2_ which, in turn, is converted into HNO_3_, H_2_O, and NO. The latter is oxidized by molecular oxygen back to NO_2_, thus justifying the higher efficiency of the NO_2_/air system if compared with anaerobic conditions.

**Scheme 10:**
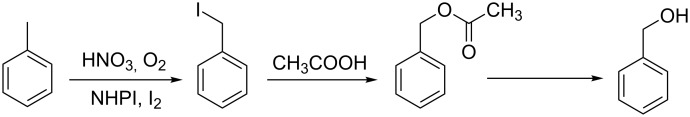
Benzyl alcohols from alkylbenzenes.

In this context, we reported in 2004 that the HNO_3_/O_2_/I_2_ system could promote the nitric aerobic oxidation of alkylbenzenes under NHPI-catalysis, leading to the selective formation of benzyl alcohols through the corresponding acetates, if operating in acetic acid solution (Scheme 10) [26]. According to the proposed mechanism, being that the concentrations of NO_2_ and O_2_ are much lower than that of I_2_, benzyl radicals generated from hydrogen abstraction by PINO react faster with the latter one, forming benzyl iodides selectively. Under the described reaction medium benzyl iodides undergo solvolysis, affording the corresponding benzyl acetates in excellent yields.

#### Enzyme laccase

Enzyme laccase is a family of “blue-copper” oxidase proteins, containing four copper ions in the active site, which cooperates in the degradation of the biopolymer lignin in woody tissues. With respect to other powerful oxidant enzymes, laccase has a lower redox potential. For this reason, its catalytic action by promoting single-electron oxidation steps is effective only on the phenolic moieties of lignin, with the concomitant reduction of oxygen to water.

**Scheme 11:**
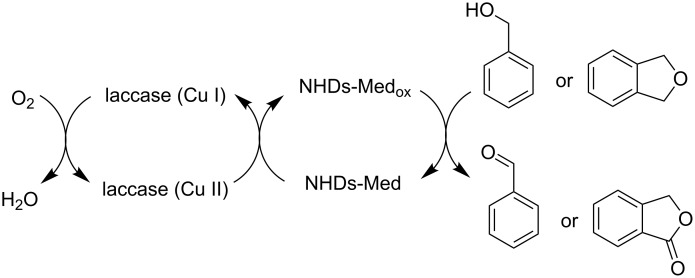
Catalytic cycle of laccase-NHDs mediator oxidizing system.

Nevertheless, the use of suitable mediators allowed the field of application of this enzyme to be extended to the catalytic oxidation of a wider range of nonphenolic substrates. Among the huge number of mediators, *N*-hydroxy derivatives (NHDs) turned out to be particularly valuable and have been widely investigated. The role of NHDs-mediators in the laccase oxidation is outlined in Scheme 11. They act as electron carriers that, once oxidized by the enzyme, diffuse in the reaction medium and in turn oxidize those organic substrates unable to enter the enzymatic pocket due to their size. Even if the activity of laccase should be ascribed to the Cu(I) inner core, the enzymatic nature of this oxidizing system allows us to consider this approach as an example of the metal-free activation of NHIs, as no further addition of transition-metal complexes is required.

**Figure 1:**
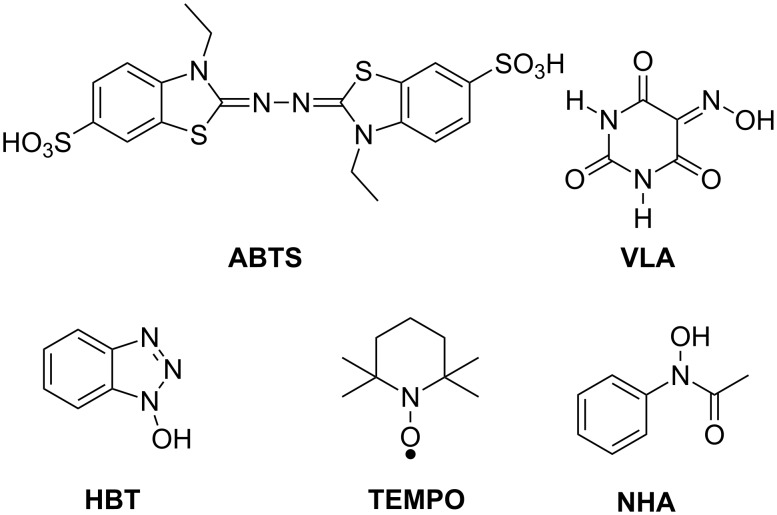
Mediators of laccase.

Galli and co-workers [27–28] significantly contributed over the years to the interpretation of the reaction mechanism by comparing NHPI and many other NHDs (Figure 1) with 2,2’-azinobis(3-ethylbenzthiazoline-6-sulfonate) (ABTS), which was the first to be used among mediators of laccase [29]. Laccase-NHDs mediator systems were successfully employed for the aerobic oxidation of nonphenolic substrates such as benzyl alcohols [27–28,30–31] and ethers [32].

Whereas laccase-ABTS follows an electron transfer (ET) mechanism, NHPI, VLA, HBT, and NHAmediators promote a hydrogen atom transfer (HAT) route through the formation of the corresponding *N*-oxyl radicals as NHDs-Med_ox_ species (Scheme 11). The same research group also emphasized the specialization of mediators versus the substrate. In fact, the laccase-TEMPO system, which operates through an ionic route by formation of oxammonium ion as NHDs-Med_ox_, resulted in particularly efficient promotion of the oxidation of benzyl alcohols, while it gave poor performances when applied in the presence of ethers. In contrast, laccase-NHPI and laccase-HBT systems, which follow a radical mechanism, showed high catalytic activity for the oxidation of ethers.

#### Quinones and analogous derivatives

**Scheme 12:**
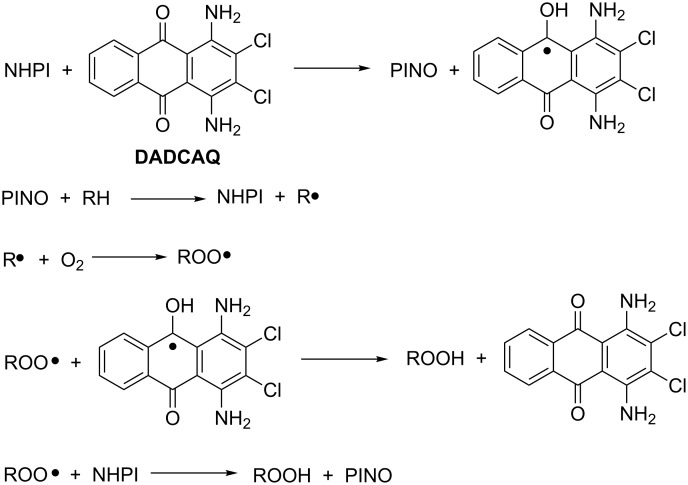
DADCAQ/NHPI-mediated aerobic oxidation mechanism.

As stressed before, the promotion of biological oxygenation is usually mediated by one-electron transfers, which lead to the formation of radicals. On the basis of this consideration, Xu and co-workers suggested that quinones, ubiquitous in nature and often involved in ET chains, could be employed to design biomimetic oxygenation models for the activation of NHPI [33]. The catalytic redox cycle is reported in Scheme 12.

The one-electron-transfer interaction of antraquinone (AQ) with NHPI in zeolite HY, followed by hydrogen-atom transfer, successfully led to the formation of the PINO radical, which in turn was responsible for the propagation of the radical chain in the selective oxidation of ethylbenzene to the corresponding acetophenone [33]. Among the different AQ derivatives that were tested, 1,4-diamino-2,3-dichloro-antraquinone (DADCAQ) was the most effective in terms of both conversion and selectivity of the ketone product.

The potential of this catalytic system was proved by extending its application, in the absence of zeolite, for the oxygenation of a wider range of hydrocarbons [34].

Moreover, the electronic effect of substituents on quinones and on the aromatic ring of NHPI was also investigated.

Quinones bearing halogen groups were used in the selective oxidation of alkylarenes, alkenes and alkanes [35], revealing how the moderate electron-withdrawing power of the substituents had a beneficial effect on the ET process. The combined activity of tetrabromo-1,4-benzoquininone (TBBQ) and NHPI afforded the best results.

**Scheme 13:**
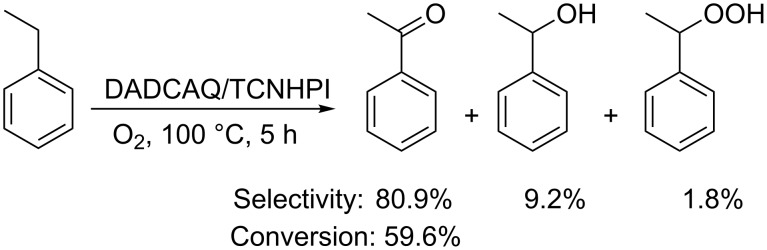
DADCAQ/TCNHPI mediated aerobic oxidation of ethylbenzene.

Aryl-tetrahalogenated NHPI derivatives were also prepared and used in combination with DADCAQ for the oxidation of ethylbenzene [36]. In particular, aryl-tetrachloro-NHPI (TCNHPI) allowed significantly higher conversion and selectivity with respect to the NHPI/DADCAQ classical system (Scheme 13).

More recently, analogous results were achieved by combining NHPI with 2,3-dichloro-5,6-dicyanobenzoquinone (DDQ) [37].

**Scheme 14:**
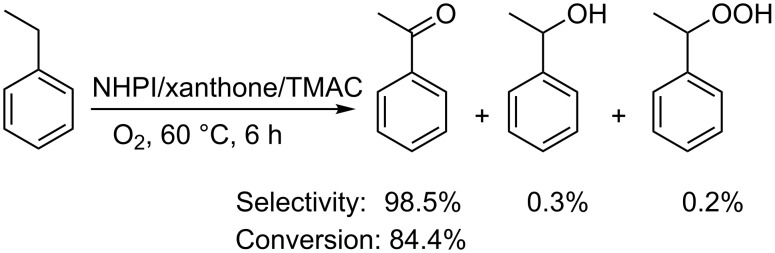
NHPI/xanthone/TMAC mediated aerobic oxidation of ethylbenzene.

Xu et al. also reported a similar one-electron-transfer activation of NHPI promoted by nonmetal xanthone and tetramethylammonium chloride (TMAC), for the selective oxidation of hydrocarbons (Scheme 14) [38]. In the proposed mechanism, TMAC has the unique role of decomposing the hydroperoxide intermediate, prolonging the free-radical chain [39–41].

**Scheme 15:**
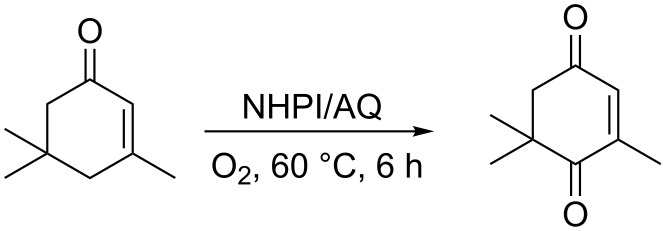
NHPI/AQ-mediated aerobic oxidation of *α*-isophorone.

The NHPI-activation by AQ has been also adopted by other research groups. Li and co-workers applied the NHPI/AQ system to promote the metal and solvent-free oxidation of α-isophorone to ketoisophorone, preventing the isomerization process of the substrate to β-isophorone (Scheme 15) [42–43].

**Scheme 16:**
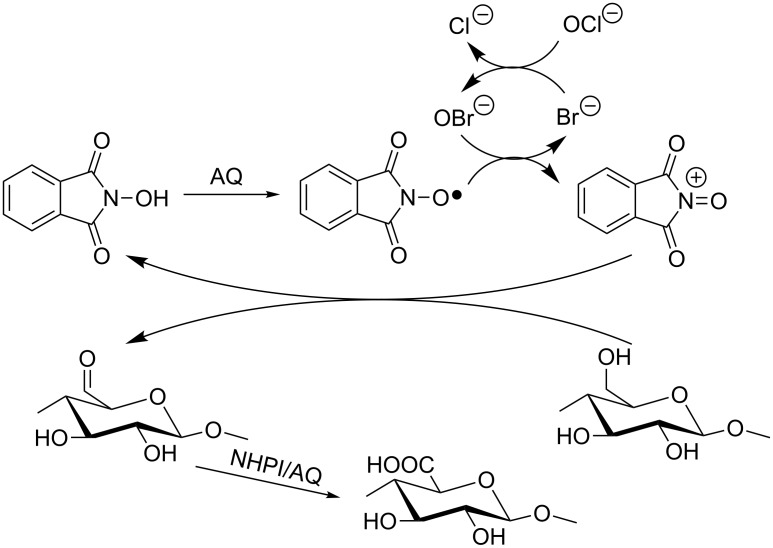
NHPI/AQ-mediated oxidation of cellulose fibers by NaClO/NaBr system.

Very recently, Coseri et al. reported that NHPI [44] and other nonpersistent nitroxyl radical precursors, such as VLA, HBT and *N*-hydroxy-3,4,5,6-tetraphenylphthalimide (TPNHPI) [45], were suitable catalysts for the selective oxidation of cellulose fibers promoted by the NaClO/NaBr system (Scheme 16). According to the proposed mechanism PINO radical is oxidized to the corresponding *N*-oxammonium cation, which in turn is responsible for the oxidation of the C6 alcoholic function.

The surface modification of cellulose fibers by selectively converting primary hydroxyl groups to the corresponding carboxylic functions, maintaining the original backbone of the polysaccharide, is of major interest for different applications [46–47].

By comparing different activation approaches, including transition-metal complexes, Coseri found that the NHPI/AQ catalytic system allowed higher conversion of hydroxyl groups. A comparison study on the effect of TEMPO and PINO radicals on the oxidation efficiency toward cellulose led to the conclusion that the NHPI/AQ oxidation mediator affords the highest content of carboxylic groups and better preserves the morphology and the molecular weight of the starting material [48].

**Scheme 17:**
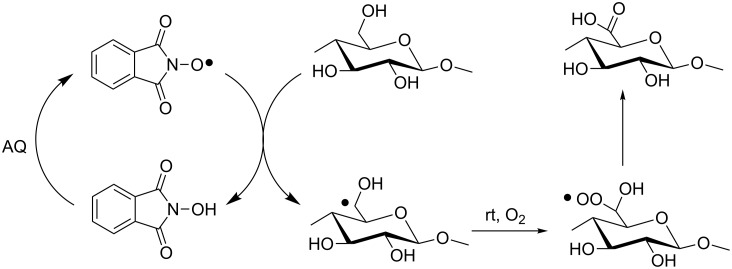
NHPI/AQ mediated aerobic oxidation of cellulose fibers.

Moreover, this catalytic system could be employed with dioxygen in place of NaClO as the ultimate oxidizing agent [49]. In this case, the mechanism follows a radical chain via classical HAT by PINO abstraction (Scheme 17).

#### Aldehydes and the molecule-induced homolysis

Sacrificial reductants have been widely reported in the literature as reactive agents able to promote the autoxidation reaction of less-reactive hydrocarbons. In this context, aldehydes have attracted increasing attention [50].

In principle, the aerobic co-oxidation promoted by aldehydes could be considered a nongreen and expensive process, due to the need for sacrificial reagents. However, this approach for oxygen activation could become competitive for practical application if the high efficiency in substrate conversion, the high selectivity in the final product, and/or the field of application, together with the low cost and environmental impact of the selected aldehyde, were significant enough to justify the reagent sacrifice. Moreover, co-oxidation of aldehydes would become attractive also if the acyl derivatives were not used as stoichiometric reagents, but just in catalytic amounts as initiators of free-radical chains.

The use of aldehydes for the activation of NHPI in an aerobic co-oxidative process was first reported by Einhorn and co-workers in 1997 [51]. The combination of stoichiometric amounts of acetaldehyde with catalytic quantities of NHPI promoted the oxidation of a wide range of hydrocarbons, including cumene and ethylbenzene, to the corresponding carbonyl groups. Under these operative conditions, peracetic acid was directly responsible for the substrate oxidation.

**Scheme 18:**
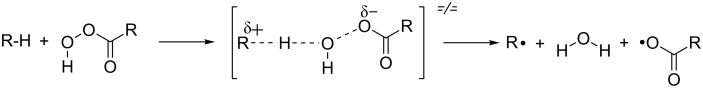
Molecule-induced homolysis by peracids.

More recently we suggested that the NHPI/aldehyde system could promote the formation of PINO radical following a molecule-induced homolysis mechanism [52]. Molecule-induced initiation is a process driven by a thermodynamic effect and consists of a bimolecular reaction according to which an OH radical, generated from hydroperoxides or peracids, undergoes hydrogen abstraction from a suitable molecule bearing relatively weak X–H bonds. The result is the formation of two radical species and a molecule of water (Scheme 18) [53–55].

We assumed that an analogous homolysis induced by peracids could occur for this NHPI, leading to the formation of PINO radical under mild and metal-free conditions.

**Scheme 19:**
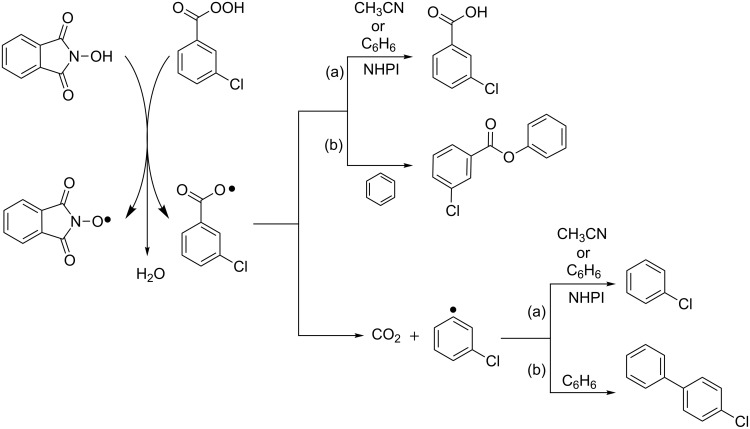
Molecule-induced homolysis of NHPI/*m*- chloroperbenzoic acid system.

This hypothesis was supported by spectroscopic and analytical evidence. By simply adding NHPI to a solution of acetonitrile containing *m*- chloroperbenzoic acid we could observe the characteristic Electron Paramagnetic Resonance (EPR) spectrum of PINO radical, while *m*-chlorobenzoic acid (90%) and Cl-benzene (10%) resulted as the unique reaction products (Scheme 19, path a) [52]. When the analogous experiment was conducted in benzene as solvent, *m*-chlorobenzoate and *m*-chlorobiphenyl were also detected (Scheme 19, path b).

On the basis of these experimental results, we suggested that the aerobic oxidation of aldehydes could be performed for the in situ generation of the corresponding peracids in the presence of NHPI, promoting co-oxidative processes catalyzed by PINO.

In an early protocol, we reported the NHPI-catalyzed selective aerobic epoxidation of α-olefins and cyclic olefins in the presence of stoichiometric amounts of aldehydes [52].

**Scheme 20:**
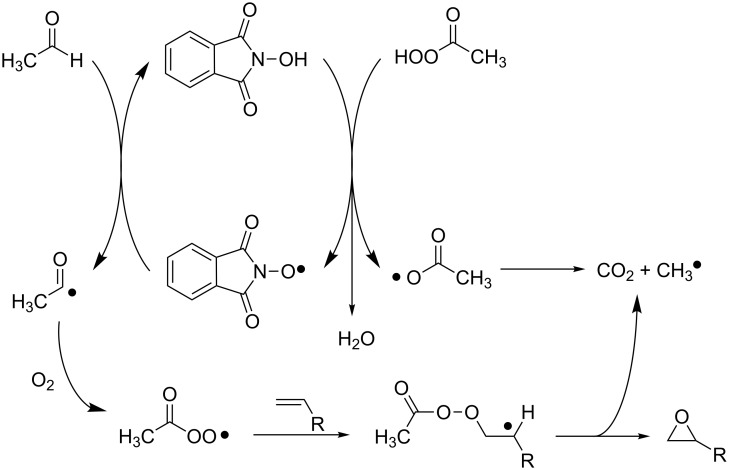
Proposed mechanism for the NHPI/CH_3_CHO/O_2_-mediated epoxidation.

The experimental results revealed an opposite selectivity with respect to classical epoxidation by peracids, with internal olefins, which were unreactive under our operating conditions. We suggested a free-radical mechanism according to which the acyl peroxyl radical generated in situ is the real epoxidizing agent (Scheme 20).

This protocol was successfully applied on a larger scale (1 liter Büchi glass vessel) for the synthesis of propylene oxide from the corresponding propene [56], and more recently under continuous-flow conditions, by means of a new multijet oscillating disk (MJOD) reactor, designed and developed by Bjørsvik and co-workers [57]. In this latter case we succeeded in accelerating the overall process, shortening the residence time with respect to the batch protocol.

On the basis of this mechanistic evidence, we also decided to investigate the Einhorn’s process for the oxidation of cumene and ethylbenzene more thoroughly, as we were expected to find a high selectivity in the corresponding hydroperoxides, which were not mentioned in the first report.

Moreover, we also assumed that high amounts of aldehyde were detrimental for the selectivity of the process, the aldehyde having the unique role of initiating the radical chain by generating PINO radical by molecule-induced homolysis.

**Scheme 21:**
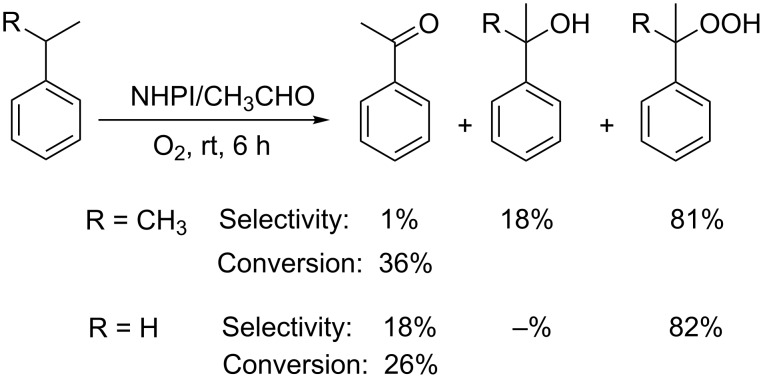
NHPI/CH_3_CHO-mediated aerobic oxidation of alkyl aromatics.

Indeed, our hypotheses were confirmed and we succeeded in increasing the selectivity in hydroperoxides up to values higher than 80% by simply reducing the amount of acetaldehyde to 10% mol ratio with respect to the alkyl aromatic [58–59] (Scheme 21).

Thus, while the NHPI/AQ catalytic system was particularly effective in converting alkylaromatics to the corresponding carbonyl derivatives, this approach represents a valuable alternative when hydroperoxides are the desired products.

Even if the presence of a polar solvent is crucial to maintain the polar catalyst in solution, it was possible to conduct the aerobic oxidation of cumene at 70 °C in the presence of 1% NHPI, 2% of acetaldehyde, and with a volume ratio cumene/CH_3_CN of 5/2, achieving the desired hydroperoxide in 28% yield with 84% of selectivity. Similarly, ethylbenzene was oxidized to the corresponding hydroperoxide with a lower yield (13%), but a higher selectivity (91%), by operating at the same temperature with 2% NHPI, 2% acetaldehyde, and with a volume ratio ethylbenzene/CH_3_CN of 1/1. These results are objects of two patent applications [60–61].

#### Light-induced activation

The first example of light-induced in situ generation of PINO radical was reported in 2007 by Lucarini and co-workers [62]. Irradiation of *N*-alkoxyphthalimides with filtered light (λ > 300 nm) from a mercury lamp promoted the selective homolysis of the O–C bond, leading to the formation of the *N*-oxyl radical, as documented by the strong characteristic EPR signal. The efficiency of the initiation approach was documented by measuring the dioxygen consumption during the aerobic oxidation of cumene. In the absence of light, cumene was completely inert, thus proving the intervention of the catalysts in the oxidative cycle.

**Scheme 22:**
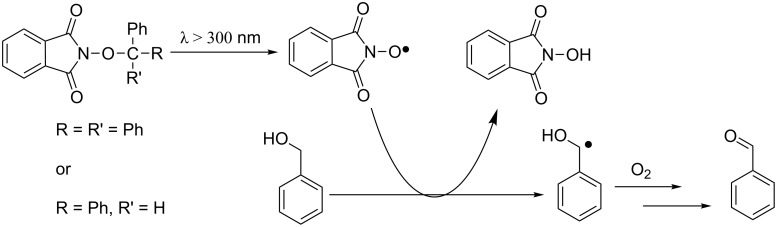
Light-induced generation of PINO from *N*-alkoxyphthalimides.

This process was successfully employed in the selective oxidation of benzyl alcohol at room temperature, affording the corresponding aldehyde in 4 h, in accordance with the results previously reported by our group operating under NHPI/Co(OAc)_2_ catalysis [63–64] (Scheme 22).

Even if this procedure seems to be particularly valuable, as no further radical mediators or initiators are required, it cannot be applied directly to NHPI.

More recently, Antonietti and co-workers have reported the photocatalytic oxidative activation of NHPI by graphitic carbon nitride (g-C_3_N_4_) and visible light irradiation [65].

g-C_3_N_4_, the most stable allotrope of carbon nitride, is a two-dimensional polymer with a tri-*s*-triazine ring unit and a *π*-conjugated layered structure similar to graphene. It is a medium-band-gap semiconductor and has proved to be an efficient photocatalyst for synthetic purposes [66–67].

It has been demonstrated that the excited state of g-C_3_N_4,_ obtained by irradiation with visible light, is able to activate O_2_ to the corresponding superoxide radical. The latter could undergo hydrogen abstraction from NHPI, leading to the generation of PINO.

**Scheme 23:**
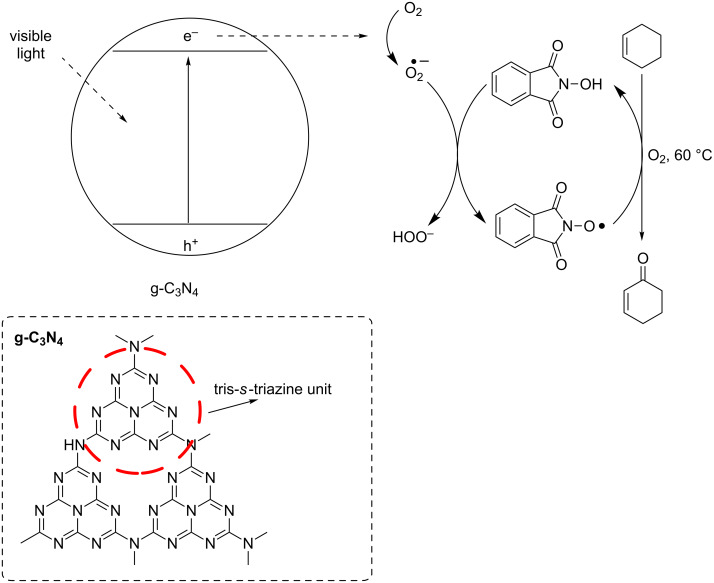
Visible-light/g-C_3_N_4_ induced metal-free oxidation of allylic substrates.

This approach was successfully applied to promote the visible-light-induced metal-free oxidation of allylic substrates (Scheme 23).

#### Other initiators

In their ongoing research dedicated to NHPI activation towards the aerobic oxidation of hydrocarbons, Xu and co-workers developed other redox initiators in addition to the NHPI/quinone systems previously described.

**Scheme 24:**
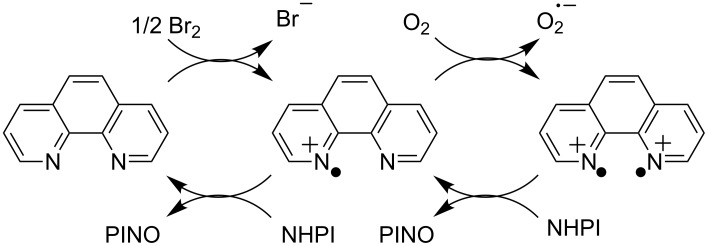
NHPI/*o*-phenanthroline-mediated organocatalytic system.

In 2005 they reported the selective oxygenation of ethylbenzene to the corresponding acetophenone by means of a NHPI/*o*-phenanthroline-mediated organocatalytic system, in the presence of molecular bromine as a co-catalyst [68] (Scheme 24).

According to the proposed mechanism, Br_2_ generates ET processes by oxidizing the *o*-phenanthroline to the corresponding cation radicals. The latter promote in turn ET and HAT processes with NHPI, leading to the formation of PINO.

In 2009 the same research group also performed the oxidation of ethylbenzene and other hydrocarbons by combining NHPI with alkaline-earth chlorides under an aerobic atmosphere [69]. The intervention of the PINO radical in the oxidation mechanism was confirmed by UV–vis and FTIR spectroscopy. MgCl_2_ was found to be the most effective salt among those tested for this purpose.

**Scheme 25:**
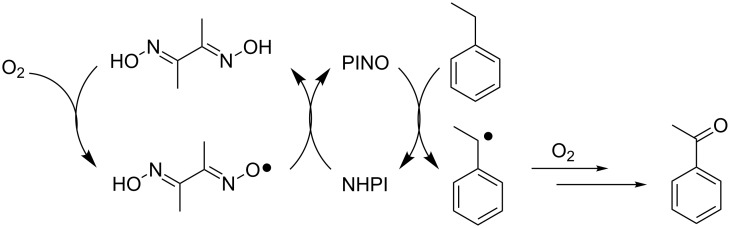
NHPI/DMG-mediated organocatalytic system.

An efficient and selective aerobic oxidation of hydrocarbons to their oxygenated products was also achieved by Zheng et al. by combining NHPI with dimethylglyoxime (DMG) [70]. The suggested mechanism is depicted in Scheme 25.

Once again all the approaches described above led to the formation of acetophenone as the major product of ethylbenzene oxidation.

A different result was achieved by Fierro et al. who, in the same year, reported the ethylbenzene oxidation to its hydroperoxide by operating in the presence of NHPI (or other NHIs, i.e., *N*-hydroxysuccinimide, *N*-hydroxymaleimide and *N*-hydroxynaphthalimide) and tiny amounts of sodium hydroxide (5 **×** 10^−3^ mol % with respect to ethylbenzene) at 421 K and 0.3 MPa [71]. The addition of NaOH determined a significant increase of selectivity in hydroperoxide (77.3%), while the ethylbenzene conversion slightly decreased from 24.5 to 21.3%.

**Scheme 26:**
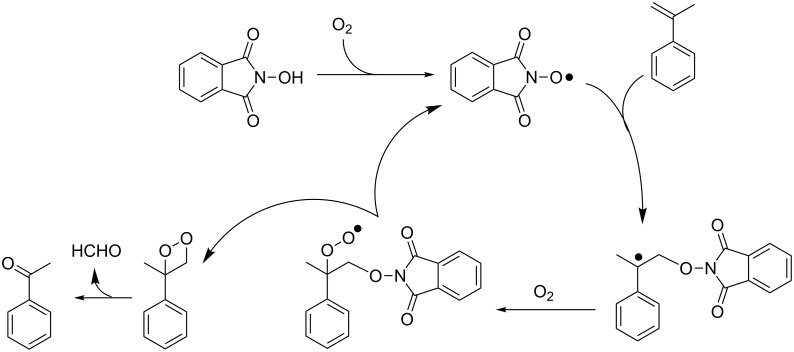
NHPI catalyzed oxidative cleavage of C=C bonds.

Very recently NHPI was reported to catalyze selective transformations without requiring additional initiators. Jiao et al. described the catalyzed oxidative cleavage of C=C double bonds using molecular oxygen as the final oxidant [72]. Following this approach α-methylstyrene was converted to acetophenone in 80% yield by operating at 80 °C in *N*,*N*-dimethylacetamide (DMA) as solvent. A plausible mechanism is shown in Scheme 26. It is based on three experimental data sets collected by the research group: (i) The occurrence of a free-radical initiation. In fact, in the presence of the radical scavenger BHT no conversion was observed. (ii) The exclusion that epoxide could be an intermediate of the process. When 2-methyl-2-phenyloxirane was used in place of α-methylstyrene, poor results were obtained in terms of yield in acetophenone. (iii) The proof that the oxygen atom in acetophenone originated from molecular oxygen, by conducting the oxygenation in the presence of ^18^O_2_.

**Scheme 27:**
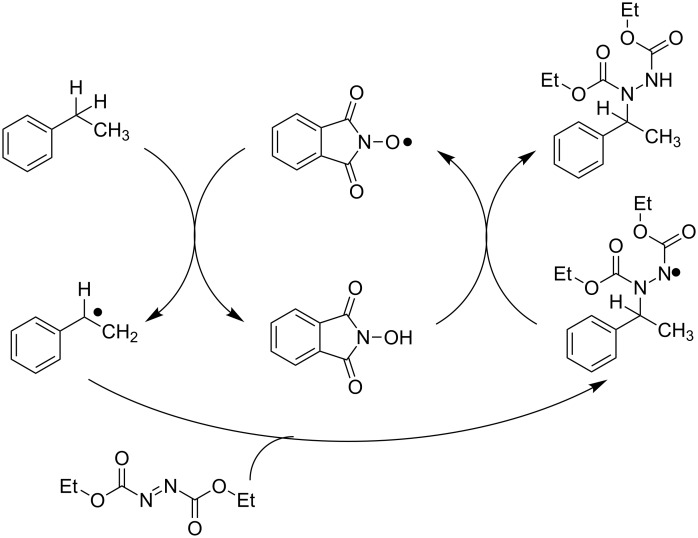
Synthesis of hydrazine derivatives.

Finally, Inoue and co-workers reported an efficient C(sp^3^)–N bond-forming method consisting of the chemoselective conversion of C(sp^3^)–H bonds in the presence of stoichiometric amounts of dialkyl azodicarboxylate reagent [73]. The proposed mechanism (Scheme 27) suggests a double function of the dialkyl azodicarboxylate, which acts both as an oxidant, promoting the oxidation of NHPI to PINO by ET process, and as acceptor of carbon radicals generated via HAT by PINO, leading to the formation of hydrazine derivatives. The scope of the reaction was investigated and its applicability was extended to different C–H bonds, including benzylic, propargylic, and aliphatic ones.

Furthermore, the hydrazine compounds were readily converted to the corresponding carbamates and amines.

## Conclusion

Metal-free oxidation catalyzed by *N*-hydroxyphthalimide and analogous *N*-hydroxy imides is becoming an essential tool for all research groups directly involved in the development of selective transformations mediated by molecular oxygen. This increasing interest is testified by several papers that have appeared in the past few years in this field and are herein discussed, describing intriguing free-radical routes for the generation of *N*-oxyl active species under mild conditions. The fields of application range from large-scale production, such as the selective oxidation of alkyl aromatics, to the synthesis of fine chemicals.

Metal-free PINO activation represents another decisive step towards the consecration of NHPI as the mediator of first choice to perform green and selective aerobic oxidations. The next one would be the engineering of valuable solutions for the complete recovery and recycle of this homogeneous organocatalyst. Once reached, this final goal, combined with the use of molecular oxygen as stoichiometric oxidant under mild operative conditions, would definitely open the way towards much more cost-effective and environmentally friendly oxidative processes.
